# Membrane Fluidity Is Regulated Cell Nonautonomously by *Caenorhabditis elegans* PAQR-2 and Its Mammalian Homolog AdipoR2

**DOI:** 10.1534/genetics.118.301272

**Published:** 2018-07-11

**Authors:** Rakesh Bodhicharla, Ranjan Devkota, Mario Ruiz, Marc Pilon

**Affiliations:** Department of Chemistry and Molecular Biology, University of Gothenburg, S-405 30, Sweden

**Keywords:** fatty acid, HEK293, lipoprotein, albumin, vitellogenin, mosaic analysis, *fat-6*, *fat-7*, *paqr-2*, *iglr-2*, adiponectin receptor

## Abstract

Maintenance of membrane properties is an essential aspect of cellular homeostasis of which the regulatory mechanisms remain mostly uncharacterized. In *Caenorhabditis elegans*, the PAQR-2 and IGLR-2 proteins act together as a plasma membrane sensor that responds to decreased fluidity by promoting fatty acid desaturation, hence restoring membrane fluidity. Here, we used mosaic analysis for *paqr-2* and *iglr-2*, and tissue-specific *paqr-2* expression, to show that membrane homeostasis is achieved cell nonautonomously. Specifically, we found that expression of *paqr-2* in the hypodermis, gonad sheath cells, or intestine is sufficient to suppress systemic *paqr-2* mutant phenotypes, including tail tip morphology, membrane fluidity in intestinal cells, cold and glucose intolerance, vitellogenin transport to the germline, germ cell development, and brood size. Finally, we show that the cell nonautonomous regulation of membrane homeostasis is conserved in human cells: HEK293 cells that express AdipoR2, a homolog of *paqr-2*, are able to normalize membrane fluidity in distant cells where AdipoR2 has been silenced. Finally, using *C. elegans* mutants and small interfering RNA against Δ9 stearoyl-CoA desaturase in HEK293 cells, we show that Δ9 desaturases are essential for the cell nonautonomous maintenance of membrane fluidity. We conclude that cells are able to share membrane components even when they are not in direct contact with each other, and that this contributes to the maintenance of membrane homeostasis in *C. elegans* and human cells.

## Abstract (precis)

The properties of cell membranes are determined mostly by the types of fatty acids that they contain. Bodhicharla *et al.* report that a key regulator of membrane fluidity, the PAQR-2/IGLR-2 protein complex...

THE *Caenorhabditis elegans* protein PAQR-2 is a member of the PAQR protein family and is homologous to the antidiabetic mammalian proteins AdipoR1 and AdipoR2 (Svensson *et al.* 2011; Devkota *et al.* 2017). Various lines of evidence, ranging from crystal structure determination to *C. elegans* genetics, suggest that PAQR-2 and its AdipoR homologs have seven transmembrane domains with their N terminus in the cytosol (Svensson *et al.* 2011; Tanabe *et al.* 2015; Vasiliauskaité-Brooks *et al.* 2017), have a hydrolases activity capable of using ceramides as substrates (Holland *et al.* 2011; Pei *et al.* 2011; Vasiliauskaité-Brooks *et al.* 2017) and are required for membrane homeostasis during cold adaptation (Svensson *et al.* 2011; Svensk *et al.* 2013) or upon a rigidifying challenge by exogenous saturated fatty acids (SFAs) (Svensk *et al.* 2016; Devkota *et al.* 2017). The *paqr-2* mutant is also intolerant of glucose because it is readily converted to membrane-rigidifying SFAs by the dietary *Escherichia coli* (Devkota *et al.* 2017). Additionally, the *paqr-2* mutant has reduced brood size, length, locomotion rate and life span, and a withered tail tip (Svensson *et al.* 2011). All of these phenotypes seem secondary to a primary membrane homeostasis defect since they are abrogated by mutations that result in increased unsaturated fatty acids (UFAs) production, by the inclusion of UFAs in the diet or by supplementation of the culture plate with membrane-fluidizing concentrations of nonionic detergents (NP-40 or Triton X-100) (Svensk *et al.* 2013, 2016; Devkota *et al.* 2017). Also, suppression of the *paqr-2* mutant phenotypes is invariably associated with improved membrane fluidity, which we have measured *in vivo* using fluorescence recovery after photobleaching (FRAP) (Svensk *et al.* 2016; Devkota *et al.* 2017).

Our previous work also identified IGLR-2 as a protein essential for PAQR-2 function: the two proteins are coexpressed strongly in the plasma membrane of the gonad sheath cells, physically interact with each other, and give identical phenotypes when mutated (except for the observation that IGLR-2 is required for high PAQR-2 expression on the gonad sheath) (Svensk *et al.* 2016). Based on a small-scale mosaic analysis, we previously showed that IGLR-2 expression in the hypodermis is sufficient to restore glucose tolerance, which suggests that IGLR-2 can act cell nonautonomously to maintain membrane fluidity systemically (Svensk *et al.* 2016). In this study, we used a more extensive mosaic analysis and tissue-specific expression studies to show that both PAQR-2 and IGLR-2 can act cell nonautonomously from several different tissues to maintain membrane fluidity throughout the worm. Additionally, we show that human cells that express AdipoR2 can remotely rescue membrane homeostasis in AdipoR2-deficient cells, suggesting that cell nonautonomous membrane homeostasis is evolutionarily conserved.

## Materials and Methods

### *C. elegans* strains and cultivation

The wild-type *C. elegans* reference strain N2, the transgene-carrying strains MD702 *{bcIs39 [lim-7p::ced-1::GFP + lin-15(+)]}*, *OD95 {ltIs37 [(pAA64) pie-1p::mCherry::his-58 + unc-119(+)]* IV; ltIs38 *[pie-1p::GFP::PH(PLC1delta1) + unc-119(+)]}*, and *RT130 [pwIs23 (vit-2::GFP)]*, and the mutant alleles studied are available from the *C. elegans* Genetics Center (Minneapolis, MN). Unless otherwise stated, experiments were performed at 20°, using the *E. coli* strain OP50 as food source, which was maintained on LB plates kept at 4° (restreaked every 6–8 weeks), and single colonies were picked for overnight cultivation at 37° in LB medium before being used to seed NGM plates (Sulston and Hodgkin 1988); new LB plates were streaked every 3–4 months from OP50 stocks kept frozen at −80°. For glucose plates, stock solutions of 1 M glucose, were filter-sterilized, and then added to cooled NGM after autoclaving. The *paqr-2(tm3410)* and *iglr-2(et34)* mutant alleles were used in most experiments and are simply referred to as the *paqr-2* and *iglr-2* mutants. The *pfat-7::GFP (rtIs30)*–carrying strain *HA1842* was a kind gift from Amy Walker (Walker *et al.* 2011).

The PHX649 (*fat-6*::*GFP*) strain was created by Suny Biotech (Fuzhou City, China) using CRISPR/Cas9 and carries a modified *fat-6* locus where the end of the coding region is fused in-frame with that of GFP. The altered sequence is as follows (underlined sequences are from the endogenous *fat-6*, linker sequences are in bold, GFP coding sequences are in regular uppercase, introns and 3′UTR are in lowercase, and the STOP codon is in italics): CATGGATGTGATATTCAACGAGGAAAATCAATCATG**AGTAAAGGAGAAGAACTTTTCACTGG**AGTTGTCCCAATTCTTGTTGAATTAGATGGTGATGTTAATGGGCACAAATTTTCTGTCAGTGGAGAGGGTGAAGGTGATGCAACATACGGAAAACTTACCCTTAAATTTATTTGCACTACTGGAAAACTACCTGTTCCATGGgtaagtttaaacatatatatactaactaaccctgattatttaaattttcagCCAACACTTGTCACTACTTTCTgTTATGGTGTTCAATGCTTcTCgAGATACCCAGATCATATGAAACgGCATGACTTTTTCAAGAGTGCCATGCCCGAAGGTTATGTACAGGAAAGAACTATATTTTTCAAAGATGACGGGAACTACAAGACACgtaagtttaaacagttcggtactaactaaccatacatatttaaattttcagGTGCTGAAGTCAAGTTTGAAGGTGATACCCTTGTTAATAGAATCGAGTTAAAAGGTATTGATTTTAAAGAAGATGGAAACATTCTTGGACACAAATTGGAATACAACTATAACTCACACAATGTATACATCATGGCAGACAAACAAAAGAATGGAATCAAAGTTgtaagtttaaacatgattttactaactaactaatctgatttaaattttcagAACTTCAAAATTAGACACAACATTGAAGATGGAAGCGTTCAACTAGCAGACCATTATCAACAAAATACTCCAATTGGCGATGGCCCTGTCCTTTTACCAGACAACCATTACCTGTCCACACAATCTGCCCTTTCGAAAGATCCCAACGAAAAGAGAGACCACATGGTCCTTCTTGAGTTTGTAACAGCTGCTGGGATTACACATGGCATGGATGAACTATACAAA*TAA*ttcgatttttatgtctggttttttgtttttatctgtac.

### Plasmids

The *pPAQR-2*, *pPAQR-2::N-GFP* (called here *Ppaqr-2::paqr-2::gfp*) (Svensson *et al.* 2011) and *pIGLR-2* (Svensk *et al.* 2016) constructs have been described elsewhere. The plasmid *pTG96* carrying the *sur-5*::*gfp(NLS)* (Yochem *et al.* 1998) was a kind gift from Professor Han (Boulder, Colorado). *pRF4*, which carries the dominant *rol-6* marker, and *pPD118.33* (plasmid no. 1596; Addgene), which carries the *Pmyo-2*::*GFP* marker, have previously been described (Mello *et al.* 1991; Davis *et al.* 2008).

For *Pelt-3*::*paqr-2:GFP*, a hypodermis-specific transgene was constructed using a Gibson assembly cloning kit (NEB, Beverly, MA) by assembly of the following two DNA fragments: 2 kb upstream regulatory sequences from *elt-3* was amplified from N2 genomic DNA using the primers 5′-ctgcggctagtttgttctctcacatttataaatcaaaagaatagaccgag-3′ and 5′-ccgattccacgtcatcttcctccatccgagcttgctgagatggctggaca-3′; and the *paqr-2* coding sequence with GFP was amplified from the plasmid *Ppaqr-2:paqr-2::GFP* using primers 5′-tgtccagccatctcagcaagctcggatggaggaagatgacgtggaatcgg-3′ and 5′-ctcggtctattcttttgatttataaatgtgagagaacaaactagccgcag-3′. The assembled plasmid was injected into N2 worms at 5 ng/μl together with 3 ng/μl *Pmyo-2*:: *GFP*.

For *Punc-54*::*paqr-2:GFP*, a muscle-specific transgene was constructed using a Gibson assembly cloning kit (NEB) by assembly of the following two DNA fragments: 2 kb upstream regulatory sequences from *unc-54* was amplified from N2 genomic DNA using the primers 5′-tcctgcagcccgggggatccgttttagattatgtcacgaa-3′ and 5′-tccacgtcatcttcctccatgatttctcgcttctttcaaa-3′; and the *paqr-2* coding sequence with GFP was amplified from the plasmid *Ppaqr-2:paqr-2::GFP* using primers 5′-atggaggaagatgacgtgga-3′ and 5′-ggatcccccgggctgcagga-3′. The assembled plasmid was injected into N2 worms at 5 ng/μl together with 3 ng/μl *Pmyo-2*:: *GFP*.

For *Pges-1*::*paqr-2:GFP*, an intestine-specific transgene was constructed using a Gibson assembly cloning kit (NEB) by assembly of the following two DNA fragments: 2 kb upstream regulatory sequences from *ges-1* was amplified from N2 genomic DNA using the primers 5′-cgaattcctgcagcccgggggatccaatattctaagcttaatgaagttta-3′ and 5′-ccgattccacgtcatcttcctccatctgaattcaaagataagatatg-3′; and the *paqr-2* coding sequence with GFP was amplified from the plasmid *Ppaqr-2:paqr-2::GFP* using primers 5′-atggaggaagatgacgtggaatcggcaacaccggcggaatcgcaaaaact-3′ and 5′-ggatcccccgggctgcaggaattcgatatcaagcttatcgataccgtcga-3′. The assembled plasmid was injected into N2 worms at 5 ng/μl together with 40 ng/μl *pRF4*.

For *Plim-7*::*paqr-2:GFP*, a gonad sheath-specific transgene was constructed using a Gibson assembly cloning kit (NEB) by assembly of the following two DNA fragments: 2 kb upstream regulatory sequences from *lim-7* was amplified from N2 genomic DNA using the primers 5′-tggaattcgcccttgtctagaatgaacatctgtatgcgaaatg-3′ and 5′-attccacgtcatcttcctccataaactgccatcggtggtt-3′; and the *paqr-2* coding sequence with GFP was amplified from the plasmid *Ppaqr-2:paqr-2::GFP* using primers 5′-atggaggaagatgacgtggaatcggcaacaccggcggaatc-3′ and 5′-tctagacaagggcgaattccagcacactggcggccgttactagtt-3′. The assembled plasmid was injected into N2 worms at 5 ng/μl together with 40 ng/μl pRF4.

For *Plim-7*::*fat-6:GFP*, a gonad sheath-specific *fat-6* transgene was constructed using a Gibson assembly cloning kit (NEB) by assembly of the following two DNA fragments: the *fat-6* coding sequence was amplified from N2 genomic DNA using primers 5′-ATGACGGTAAAAACTCGTTC-3′ and 5′-ATCCAGTTCTTGAACGGTAT-3′; and the *lim-7* promoter with vector sequence was amplified from *Plim-7*::*paqr-2:GFP* plasmid using primers 5′-ATACCGTTCAAGAACTGGATAAGGGCGAATTCTGCAGATA-3′ and 5′–TTGAACGAGTTTTTACCGTCATAAACTGCCATCGGTGGTT-3′. The assembled plasmid was verified by sequencing. The plasmid was injected into *fat-6* mutant worms at 5 ng/µl together with 40 ng/µl *pTG96*.

For *Pelt-3*::*fat-6:GFP*, a hypodermis-specific *fat-6* transgene was constructed using a Gibson assembly cloning kit (NEB) by assembly of the following two DNA fragments: the *fat-6* coding sequence was amplified from N2 genomic DNA using primers 5′-ATGACGGTAAAAACTCGTTC-3′ and 5′–CCCCGGGCGTTCCGGTTTGC-3′; and the *elt-3* promoter with vector sequence was amplified from *Pelt-3*::*paqr-2:GFP* plasmid using primers 5′-GCAAACCGGAACGCCCGGGGTTATAAATCAAAAGAATAGA-3′ and 5′–GAACGAGTTTTTACCGTCATCCGAGCTTGCTGAGATGGCT-3′. The assembled plasmid was verified by sequencing. The plasmid was injected into *fat-6* mutant worms at 5 ng/µl together with 40 ng/µl *pTG96*.

### Mosaic analysis

The plasmid *pTG96* carrying the *sur-5*::*gfp(NLS)* was co-injected into the gonad syncytium of wild-type worms at a concentration of 50 ng/μl together with 5 ng/μl of *pIGLR-2* or *pPAQR-2* to establish a transgenic line. The extrachromosomal array was then crossed into the *iglr-2* or *paqr-2* mutant background, and these transgenic worms were bleached and their eggs allowed to hatch overnight in M9 to produce synchronized L1s that were transferred to NGM plates containing 20 mM glucose. Worms that grew to into adults were scored 72 hr later.

### Growth and tail tip scoring assays

For length measurement studies, synchronized L1s were plated onto test plates seeded with *E. coli*, and worms were mounted then photographed 72 hr later. The length of >20 worms was measured using ImageJ (Schneider *et al.* 2012). Quantification of the withered tail tip phenotype was done on synchronous 1-day-old adult populations, *i.e.*, 72 hr post L1 (*n* ≥ 100) (Svensk *et al.* 2013).

### FRAP in *C. elegans* and HEK293 cells

FRAP experiments in *C. elegans* were carried out using a membrane-associated prenylated GFP reporter (*pGLO-1P*::*GFP-CAAX*) expressed in intestinal cells, as previously described, using a Zeiss LSM700inv laser scanning confocal microscope with a 40× water immersion objective (Mörck *et al.* 2009; Svensk *et al.* 2016; Devkota *et al.* 2017). For FRAP in mammalian cells, HEK293 cells were stained with BODIPY 500/510 C1, C12 (4,4-difluoro-5-methyl-4-bora-3a,4a-diaza-*s*-indacene-3-dodecanoic acid; BODIPY-C12) (Invitrogen, Carlsbad, CA). FRAP images were acquired with an LSM880 confocal microscope equipped with a live cell chamber (set at 37° and 5% CO_2_) and ZEN software (Zeiss, Thornwood, NY) with a 40× water immersion objective as previously described (Devkota *et al.* 2017).

### Vitellogenin assay

Vitellogenin was analyzed in N2, *paqr-2*, and *iglr-2* worms using the *pwIs23 (vit-2::GFP)* transgene. Synchronized *vit-2*::*GFP*, *paqr-2*; *vit-2*::*GFP* and *iglr-2*; *vit-2*::*GFP* worms were spotted on control plates and incubated at 20°. After 72 hr of incubation at 20°, they were washed off the plate and mounted on agarose pads, and then observed with a Zeiss Axioskop microscope at 400× magnification. Worms were scored based on wild type and defective vitellogenin accumulation (*n* ≥ 100).

### Germline morphology

Germline morphology was analyzed in N2, *paqr-2*, and *iglr-2* worms using 4′,6′-diamidino-2-phenylindole hydrochloride (DAPI) staining. Synchronized worms were spotted on control plates and after 96 hr of incubation at 20°, they were washed and fixed with (−20°) methanol for 5 min. The supernatant was then removed and the worms washed twice with PBST. The fixed samples were then stained in 100 ng/ml DAPI in PBST for 30 min, washed twice with PBST and mounted on agarose pads, and then observed and photographed using a Zeiss Axioskop microscope.

### Germline apoptosis

Apoptotic cells was analyzed in N2, *paqr-2*, and *iglr-2* worms using the *bcIs39* *[Plim-7*::*ced-1*::*gfp*; *lin-15(+)]* transgene (Zhou *et al.* 2001). Synchronized *bcIs39*, *paqr-2;bcIs39* and *iglr-2*; *bcIs39* worms were spotted on control plates and incubated at 20°. After 72 hr of incubation at 20°, they were washed off the plate and mounted on agarose pads, and then observed and photographed using a Zeiss Axioskop microscope. Worms were scored by counting the number of apoptotic cells in the germline (*n* ≥ 20).

### Germline membrane morphology

Germline membrane morphology was analyzed in N2, *paqr-2*, and *iglr-2* worms using the. *ltIs38 [pie-1p*::*GFP*::*PH(PLC1delta1) + unc-119(+)]* transgene (McNally *et al.* 2006). Synchronized worms were spotted on control plates and incubated at 20°. After 72 hr of incubation at 20°, they were washed off the plate and mounted on agarose pads, and then observed and photographed using a Zeiss Axioskop microscope.

### Cultivation of HEK293

HEK293 were grown in DMEM containing glucose 1 g/liter, pyruvate, and GlutaMAX and supplemented with 10% fetal bovine serum, 1% nonessential amino acids, HEPES 10 mM, and 1% penicillin and streptomycin (all from Life Technologies) at 37° in a water-humidified 5% CO_2_ incubator. Cells were subcultured twice a week at 90% confluence. Cells were cultivated on treated plastic flask and multidish plates (Nunc). For FRAP experiments, HEK293 were seeded in μ-dishes (35 mm, high) containing culture-insert four wells (Ibidi) and precoated with 0.1% porcine gelatin (Sigma, St. Louis, MO).

### Small interfering RNA in HEK293 cells

The following predesigned small interfering RNAs (siRNAs) were purchased from Dharmacon: AdipoR2 J-007801-10, nontarget D-001810-10 and Δ9 stearoyl-CoA desaturase (SCD) J-005061-07. Transfection of 25 nM siRNA was performed in complete media using Viromer Blue according to the manufacturer’s instructions “1X” (Lipocalyx). Knockdown gene expression was verified 48 hr after transfection.

### Quantitative PCR in HEK293 cells

Total cellular RNA was isolated using an RNeasy Kit according to the manufacturer’s instructions (Qiagen, Valencia, CA) and quantified using a NanoDrop spectrophotometer (ND-1000; Thermo Scientific). Complementary DNA was obtained using a RevertAid H Minus First Strand cDNA Synthesis Kit (Thermo) with random hexamers. Quantitative PCR was performed with a CFX Connect thermal cycler (Bio-Rad, Hercules, CA) using Hot FIREpol EvaGreen qPCR SuperMix (Solis Biodyne) and standard primers. Samples were measured as triplicates. The relative expression of each gene was calculated according to the ΔΔCT method (Livak *et al.* 2001). Expression of the housekeeping gene PPIA was used to normalize for variations in RNA input. Primers used were as follows: AdipoR2, forward (TCATCTGTGTGCTGGGCATT) and reverse (CTATCTGCCCTATGGTGGCG); PPIA, forward (GTCTCCTTTGAGCTGTTTGCAG) and reverse (GGACAAGATGCCAGGACCC); and SCD, forward (TTCGTTGCCACTTTCTTGCG) and reverse (TGGTGGTAGTTGTGGAAGCC).

### HEK293 fatty acid treatment

Palmitic acid (PA; Sigma) was dissolved in sterile DMSO (Sigma) then mixed with fatty acid-free BSA (Sigma) in serum-free medium for 20 min at room temperature. The molecular ratio of BSA to fatty acid was 1–2.65 (when using 200 μM PA) or 1–5.3 (when using 400 μM PA). Cells were then cultivated in this serum-free media containing the fatty acids for 24 hr prior to FRAP analysis.

### Laurdan dye measurement of membrane fluidity in HEK293 cells

Live HEK293 cells were stained with Laurdan dye (6-dodecanoyl-2-dimethylaminonaphthalene) (Thermo Fisher) at 10 μM for 45 min. Images were acquired with an LSM880 confocal microscope equipped with a live cell chamber (set at 37° and 5% CO_2_) and ZEN software (Zeiss) with a 40× water immersion objective. Cells were excited with a 405 nm laser and the emission recorded between 410 and 461 nm (ordered phase) and between 470 and 530 nm (disordered phase). Pictures were acquired with 16 bits image depth and 1024 × 1024 resolution, using a pixel dwell of ∼1.02 μsec. Images were analyzed using ImageJ software (Schneider *et al.* 2012), following published guidelines (Owen *et al.* 2011).

### HEK293 transwell plate experiments

HEK293 cells were grown in transwell permeable supports (polyester membrane, 0.4 μm pore size) (Costar, Cambridge, MA) and are referred to as donor cells in the text. Then, medium in the upper chamber was replaced by serum-free medium supplemented with 0.5% BSA and [9,10-^3^H(N)]-palmitic acid (Perkin Elmer, Norwalk, CT) at a specific activity of 2 μCi for 4 hr. The medium in the lower was serum-free medium. Media were collected (loading medium), the donor cells were washed with serum-free medium and then moved to new wells preseeded with HEK293 cells (acceptor cells) and cultured with serum-free medium with ±0.5% BSA or 10% fetal bovine serum. Finally, after 24 hr, the transferring media was collected and cells were recovered with TrypLE. Sample activity was measured with a TRI-CARB 4810TR 110 V Liquid Scintillation Counter (Perkin Elmer).

### Statistics

Error bars for worm length measurements show the SEM, ANOVA and Dunnett’s multiple comparisons test were used to identify significant differences between worm lengths. Error bars for the frequency of the tail tip defect show the 95% confidence interval and significant differences determined using *Z*-tests. *t*-tests were used to determine significance in FRAP experiments. All experiments were repeated at least twice with similar results. Asterisks are used in the figures to indicate various degrees of significance, as follows: * *P* < 0.05, ** *P* < 0.01, *** *P* < 0.001.

### Data availability

The complete mosaic analysis data are presented in the Supplemental Material, Table S1, and three figures are provided as supplemental materials (Figures S1–S3). Supplemental material available at Figshare: https://doi.org/10.25386/genetics.6608843.

## Results

### Mosaic analysis identifies sufficient sites of action for *paqr-2* and *iglr-2*

**Figure 1:**
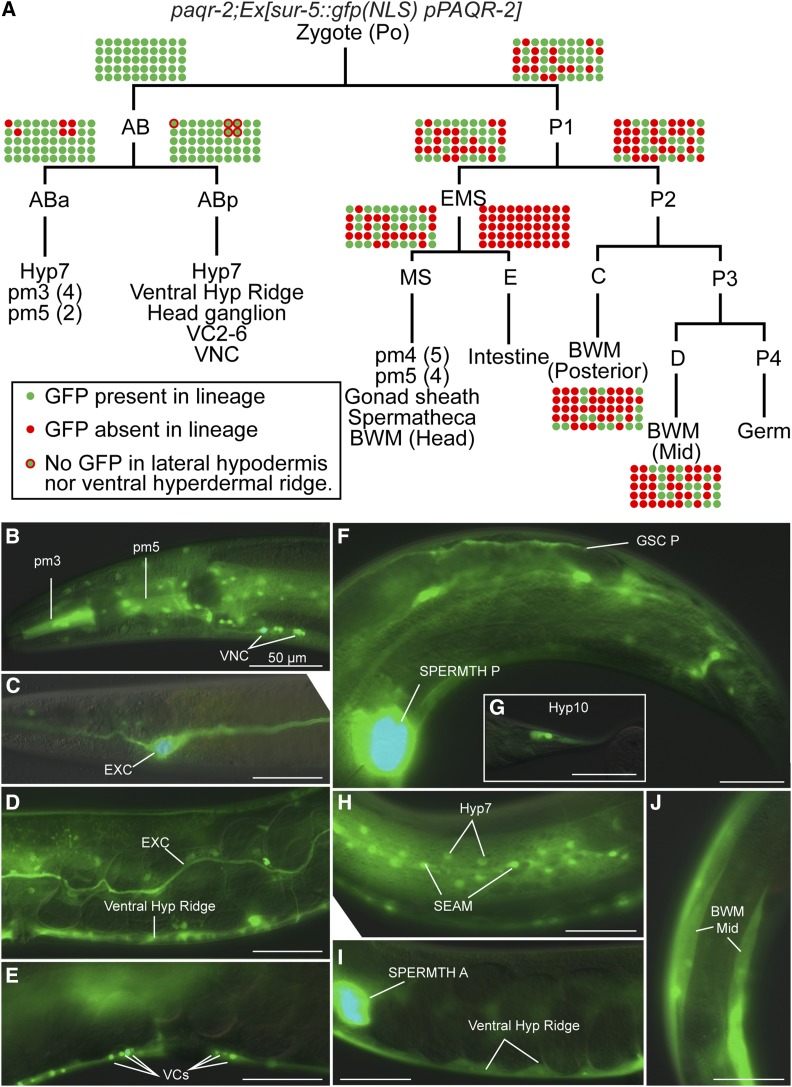
Mosaic analysis of *paqr2;Ex[sur-5*::*gfp(NLS) pPAQR-2]* worms selected for growth to adulthood on 20 mM glucose. (A) Cell lineages and tissues carrying the extrachromosomal arrays were identified by the expression of GFP. Fifty worms able to grow into adults on 20 mM glucose and not expressing GFP in the intestine were selected for the analysis because expression in that organ obscures expression elsewhere (Yochem *et al.* 1998). The transgenic statue of each cell lineage is color-coded in each of the 50 worms based on GFP expression in identifiable cells. (B–J) Example of GFP-positive tissues from a mosaic analysis study. Each panel is from a different animal carrying the *paqr-2;Ex[sur-5*::*gfp(NLS) pPAQR-2]* in different tissues, as indicated. Bars, 50 μm. BWM, body wall muscles; EXC, excretory canal cell; GSC, gonad sheath cells; SEAM, seam cells; SPERMTH A and P, anterior and posterior spermatheca, respectively; VCs, ventral cord neurons 2–6; Ventral Hyp Ridge, ventral hypodermal ridge; VNC, other ventral cord neurons.

We performed an extensive mosaic analysis to identify tissues where expression of *paqr*-2 and *iglr-2* are sufficient to rescue the mutant phenotypes. In *C. elegans*, transgenes are typically retained as extrachromosomal arrays that are not always segregated to both daughter cells during cell division, resulting in genetic mosaics. If the array carries a GFP marker, one can easily determine which cells are transgenic in a mosaic individual and later correlate sites of expression with phenotypes (Yochem *et al.* 1998). The results of our mosaic analyses show that expression of *paqr-2* and *iglr-2* in either hypodermis or gonad sheath cells is sufficient to suppress the glucose sensitivity of the mutants (Figure 1, Figure S1, and Table S1). In particular, five worms that had *paqr-2* expression in gonad sheath cells had no expression in the hypodermis, lacking the extrachromosomal transgene from the ABp lineage and carrying it in just a few nonhypodermal cells of the ABa lineage (Figure 1A). These worms were most likely rescued because of the expression of *paqr-2* in gonad sheath cells, where both PAQR-2 and IGLR-2 are predominantly expressed (Svensk *et al.* 2016). In many other mosaic *paqr-2* and *iglr-2* mutant worms, expression of the corresponding wild-type transgene was entirely lost from the P1 lineage but retained in hypodermal cells of the AB lineage, suggesting that expression in the hypodermis is also sufficient to rescue the mutant.

### Tissue-specific expression confirms the cell nonautonomous activity of *paqr-2*

**Figure 2:**
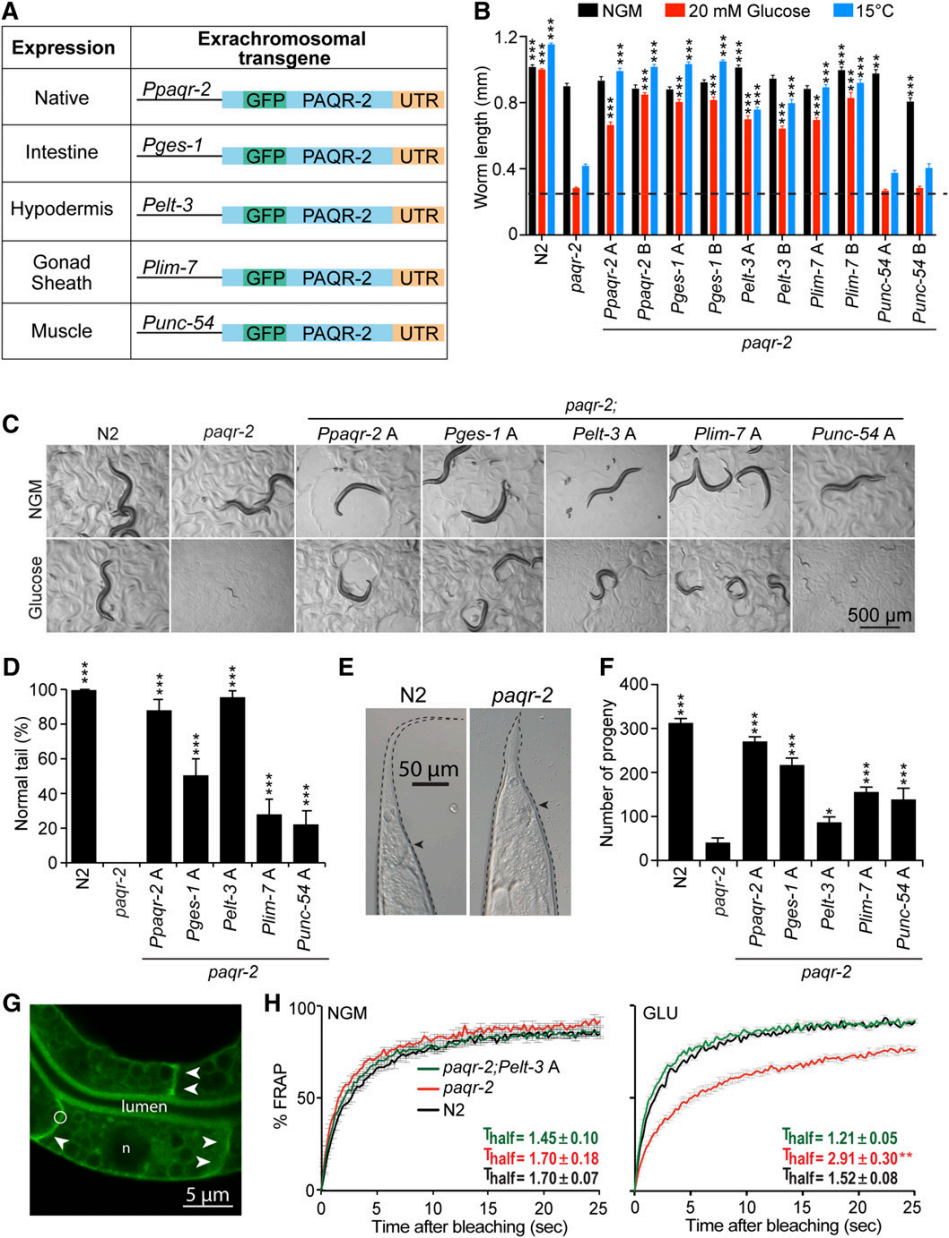
Tissue-specific rescue of *paqr-2* mutant phenotypes. (A) Overview of the constructs used and their expression patterns. (B) Average length of ≥20 worms (initially synchronized L1s) with the indicated genotypes cultivated for 72 hr on normal plates (NGM), plates containing 20 mM glucose, or at 15°. Two transgenic lines, designated “A” and “B,” were obtained for each extrachromosomal array transgene and transgenic worms were identified via the co-injected *rol*-6 marker, which causes a Roller phenotype. The dashed line represents the approximate length of the L1s at the start of the experiments, and representative images are shown in C. (D) Scoring of the tail tip phenotype in worms of the indicated genotypes, with an example of a normal and a defective tail shown in E, where the arrowhead indicates the position of the anus. At least 100 1-day-old adult worms were scored for each genotype. (F) Average brood size of 10 worms of the indicated genotypes. (G) Illustration of a FRAP experiment, showing a *pGLO-1P*::*GFP-CAAX*–positive intestinal membrane before. The circle indicates the size of the area to be bleached; arrowheads point to well-defined intestinal membranes and “n” indicates a nucleus. (H) FRAP result showing that N2, *paqr-2* mutant, and *paqr-2* mutant carrying a hypodermis-specific *paqr-2(+)* transgene have all similar membrane fluidity on normal plates (NGM), and that the hypodermis-specific expression of *paqr-2(+)* suppresses the loss of fluidity in the intestine of the *paqr-2* mutant grown on 20 mM glucose (GLU). The T_half_ is the time in seconds required to reach half of the maximum fluorescence recovery (L1+16 hr worms; *n* ≥ 5 worms). Values in B, D, and F that differed significantly from *paqr-2* are indicated. ** *P* < 0.01 and *** *P* < 0.001.

We were intrigued by the possibility that localized expression of *paqr-2* could rescue systemic phenotypes cell nonautonomously. To explore this further, we generated several tissue-specific *paqr-2* expression transgenes (Figure 2A) and scored their ability to suppress *paqr-2* mutant phenotypes. We found that *paqr-2* expression in either hypodermis or gonad sheath cells is sufficient to suppress glucose toxicity (Figure 2, B and C), cold intolerance (Figure 2B), tail morphology (Figure 2, D and E), and brood size (Figure 2F). We also found that expression of *paqr-2* specifically in the intestine itself (which could not be scored in our mosaic analysis because it obscures expression in other tissues; Yochem *et al.* 1998) could also rescue these phenotypes (Figure 2, A–E). Importantly, membrane fluidity in the intestine was normalized by hypodermis-specific *paqr-2* expression (Figure 2, G and H). Altogether these results provide very strong evidence that *paqr-2* can act cell nonautonomously to support systemic membrane homeostasis in *C. elegans*.

Expression of *paqr-2* in body wall muscles partially rescued some mutant phenotypes, including tail morphology and brood size (Figure 2, A–E), suggesting that even this tissue is able to provide some systemic benefits to membrane homeostasis. However, muscle-specific expression was not able to suppress glucose intolerance and cold sensitivity, which is consistent with the mosaic analysis results (Figure 1A).

### *fat-6* is required for *paqr-2* function in the hypodermis

**Figure 3:**
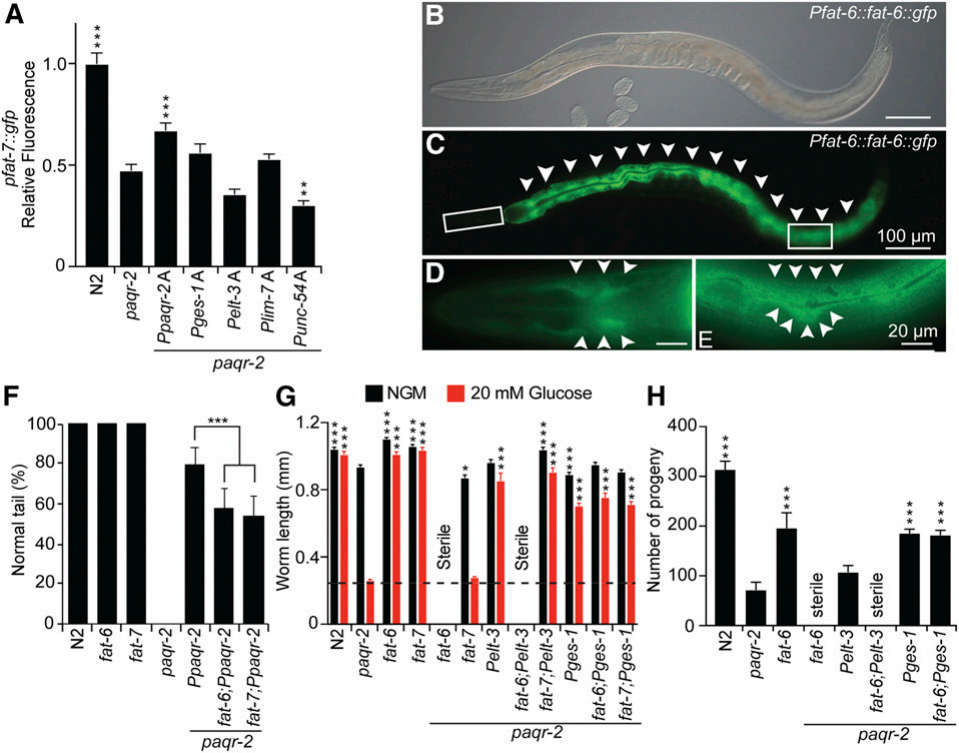
Tissue specificity of the *fat-6* and *fat-7* requirements. (A) Only *paqr-2(+)* expressed from its native promoter can significantly restore the expression levels of the *fat-7*::*gfp* reporter in the *paqr-2* mutant (1-day-old adult worms; *n* ≥ 20). (B–E) Expression pattern of the CRISPR-modified *fat-6* locus fused to GFP at the 3′ end of the coding region. Note the strong intestinal expression (arrowheads in C) as well as the distinct hypodermal cell expression in a different focal plane shown in the enlarged insets in D and E (arrowheads). (F) Tail tip phenotype of 1-day-old transgenic worms with the indicated genotypes. Both *fat-6* and *fat-7* contribute to the ability of the *Ppaqr-2(+)* transgene to suppress the tail tip phenotype of the *paqr-2* mutant (*n* ≥ 100). (G) Length of 1-day-old adult worms with the indicated genotypes cultivated on normal plates (NGM) or 20 mM glucose (*n* ≥ 20). (H) Brood size of worms with the indicated genotypes cultivated on normal plates (*n* = 10). * *P* < 0.05, ** *P* < 0.01, and *** *P* < 0.001.

The primary defect in *paqr-2* mutants appears to be an inability to enhance fatty acid desaturation when cellular membrane becomes too rigid, and Δ9 desaturases appear especially important for this process because many *paqr-2* suppressor mutations act by promoting their activity (Svensk *et al.* 2013). In particular, we previously showed that the *paqr-2* mutant has reduced expression of the intestinal Δ9 desaturase *Pfat-7*::*GFP* reporter (Svensk *et al.* 2013), which we again confirmed in this study (Figure 3A). Of all the transgenes tested, only *paqr-2* driven from its own promoter was able to significantly raise the levels of *Pfat-7*::*GFP* expression (Figure 3A). This is somewhat surprising given that these same tissue-specific *paqr-2* transgenes are able to at least partially suppress most *paqr-2* phenotypes. There are three Δ9 desaturases in *C. elegans*, namely *fat-5*, *fat-6*, and *fat-7*, and these show functional redundancies: single mutants are phenotypically wild type and only the triple mutant is lethal (Brock *et al.* 2007). *fat-6* and *fat-7* share a very high degree of sequence identity, predominantly carry out the desaturation of stearate (18:0), and their inhibition using RNA interference causes a significant reduction in *de novo* lipogenesis accompanied by reduced membrane turnover (Dancy *et al.* 2015). Both *fat-6* and *fat-7* are required for the suppression of *paqr-2* phenotypes by mutations that promote fatty acid desaturation (Svensk *et al.* 2013). However, at least two differences exist between *fat-6* and *fat-7*: *fat-6* is expressed in both the intestine and hypodermis (Brock *et al.* 2006) (Figure 3, B–E), whereas *fat-7* expression seems restricted to the intestine (Brock *et al.* 2006); and the *paqr-2;fat-6* double mutant is viable but sterile whereas the *paqr-2;fat-7* double mutant is viable and fertile (Svensson *et al.* 2011). Since we did not observe upregulation of *Pfat-7*::*GFP* in the *paqr-2* mutants rescued with various tissue-specific *paqr-2* transgenes, we decided to explore the possibility that rescue of *paqr-2* may be dependent on *fat-6*. We found that *paqr-2* mutant worms carrying the wild-type *Ppaqr-2* construct on an extrachromosomal array show a weaker suppression of the tail tip defect when either *fat-6* or *fat-7* is mutated, implicating both genes as *paqr-2* effectors (Figure 3F). Next, we found that hypodermis-specific expression of *paqr-2* is synthetic sterile with the *fat-6* but not with the *fat-7* mutation when the worms are challenged with 20 mM glucose (Figure 3G). Similarly, expression of *paqr-2* specifically in gonad sheath cells rescues the growth of *paqr-2* or *paqr-2;fat-7* mutants on normal plate and plates containing glucose, but does not rescue fertility of the *paqr-2;fat-6* double mutant (Figure S2, A and B). In contrast, intestine-specific expression of *paqr-2* suppresses the glucose intolerance of the *paqr-2* mutant even when either *fat-6* or *fat-7* is mutated (Figure 3G). *fat-6* was also not required for the suppression of the brood size defect by intestine-specific expression of *paqr-2* (Figure 3H). These results show that *fat-6* is required for the ability of hypodermis-specific expression of *paqr-2* to rescue the tail and glucose tolerance, but dispensable for the intestine-specific expression of *paqr-2* to rescue these traits as well as brood size. The simplest interpretation is that hypodermis-specific or gonad sheath cell-specific expression of *paqr-2* requires *fat-6* to provide systemic phenotypic rescue. Intestine-specific expression of *paqr-2* is not critically dependent on either *fat-6* or *fat-7* because both are expressed and functionally redundant in that tissue (Brock *et al.* 2006).

We used tissue-specific expression of *fat-6* to further investigate its site of action. In these experiments, we found that hypodermis-specific expression of both *paqr-2* and *fat-6*, driven by the *elt-3* promoter is sufficient to suppress the infertility, glucose intolerance, and the tail tip defects in *paqr-2*;*fat-6* double mutant (Figure S2, C–E). On the other hand, gonad sheath cell-specific expression of *paqr-2* and *fat-6* did not rescue the fertility defect of the *paqr-2;fat-6* double mutant (Figure S2, D and E). Altogether, these results suggest that *paqr-2*/*fat-6*-dependent lipid metabolism in the hypodermis is sufficient to rescue the worms systemically but that the gonad sheath cells may have different requirements to fulfill this function.

### Vitellogenin transport defects in the *paqr-2* and *iglr-2* mutant

**Figure 4:**
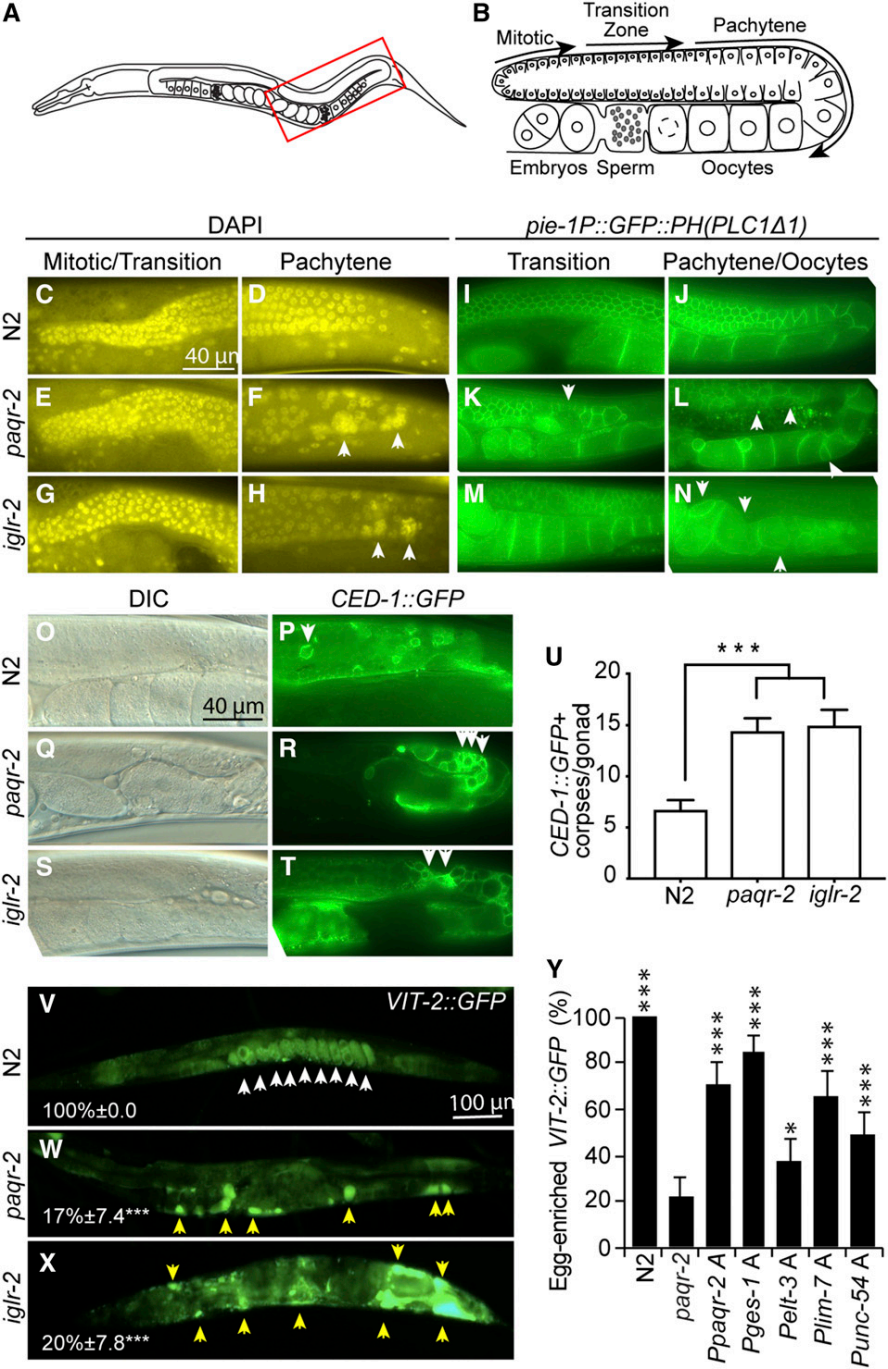
Germline and vitellogenin transport defects in the *paqr-2* and *iglr-2* mutants. (A and B) Main features of the posterior gonad of *C. elegans* (boxed area in A is detailed in B). The gonad is enclosed by the gonad sheath cells. In the distal end, nuclei share a common cytoplasm, hence forming a syncytium, and divide by mitosis and, more proximally, by meiosis. They accumulate yolk during the pachytene stage at the end of which they become fully surrounded by a membrane, becoming oocytes that continue to mature by the accumulation of yolk material. (C–H) DAPI staining showing that the *paqr-2* and *iglr-2* mutants have gross defects in their germline, especially in the pachytene region where large aggregates are evident (arrowheads). Note that the blue DAPI hue was converted to yellow to improve contrast. (I–N) Visualization of germline membranous structures reveals severe defects in germ cell shape and organization in the *paqr-2* and *iglr-2* mutants, which is most pronounced in pachytene and oocyte stages. Note the presence of deformed and abnormally small oocytes in the ventral side of the gonad in both mutants (arrowheads). (O–T) DIC images accompanied by the visualization of apoptotic corpses using CED-1::GFP reveals an increase in the frequency of cell death within the germline of the *paqr-2* and *iglr-2* mutants, (U) which is quantified in 1-day-old adult worms (*n* = 20). Note again the abnormal presence of apoptotic clusters in the mutant (arrowheads). (V–X) Visualization of the VIT-2::GFP reporter reveals a clear accumulation of vitellogenin in embryos of wild-type N2 worms (white arrowheads) whereas the *paqr-2* and *iglr-2* mutants exhibit a gross mislocalization of vitellogenin, which accumulates in the pseudocoelomic space (yellow arrowheads); the average percentage of individuals with clear egg-enriched VIT-2::GFP are indicated together with the range corresponding to their 95% confidence interval. (Y) Tissue-specific expression of *paqr-2(+)* suppressed the vitellogenin mislocation defect of the *paqr-2* mutant (see Figure 2A for a description of the transgenes used); values that differed significantly from *paqr-2* are indicated.

The above results suggest that the hypodermis, and perhaps other tissues as well, can produce UFAs that are throughout the worm to achieve cell nonautonomous and systemic membrane homeostasis. One key lipid transport mechanism in *C. elegans* is that of the vitellogenins, which are lipoprotein-like proteins secreted into the pseudocoelom by the intestine, transported through the gonad sheath cells and ultimately endocytosed by maturing oocytes via receptors of the LDL receptor superfamily such RME-2 (Kimble and Sharrock 1983; Grant and Hirsh 1999; Hall *et al.* 1999). We therefore explored a possible connection between the functions of *paqr-2* and *iglr-2* in membrane homeostasis and the lipid transport roles of vitellogenins. A careful examination of the gonads revealed that the *paqr-2* and *iglr-2* mutants have an abundance of abnormal germ cell nuclei in the pachytene zone (Figure 4, A–N), and an excess of apoptotic bodies (Figure 4, O–U) in the germline (which could be due to excess apoptosis or defects in phagocytosis of apoptotic corpses). This is accompanied by a dramatic accumulation of vitellogenins in the pseudocoelom of the mutants (Figure 4, V–Y); this defect in lipid transport likely contributes to the low brood size and increased germline apoptosis in these mutants. Note that there are no visible germline defects in *paqr-2* mutant worms at the L4 stage, *i.e.*, before yolk production begins (Figure S2F). Importantly, and just as with the intestinal membrane fluidity defect, the vitellogenin uptake defect in the *paqr-2* mutants can also be rescued cell nonautonomously by expression of *paqr-2* specifically in intestine, hypodermis, or muscle, or cell-autonomously by expression of *paqr-2* in the gonad sheath cells. We conclude that at least some forms of lipid transport, namely the transport of vitellogenins into the gonads, depends on the function of *paqr-2* and *iglr-2* and may contribute to the phenotypes of these mutants.

### Cell nonautonomous membrane homeostasis is conserved in human cells

**Figure 5:**
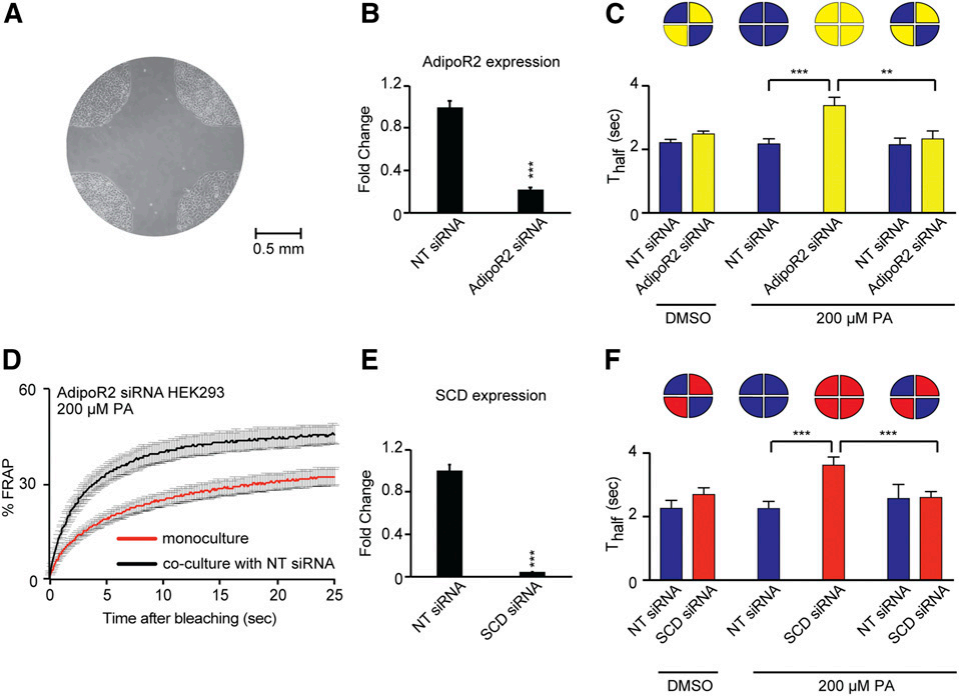
AdipoR2-positive HEK293 cells contribute to membrane homeostasis in distant cells. (A) Photograph of the center of a partitioned culture dish, after removal of the partition. Note the HEK293 cells growing in four-well separated quadrants. (B) Quantitative PCR showing that AdipoR2 siRNA lowers AdipoR2 messenger RNA levels to <20% of that in the sample treated with nontarget (NT) siRNA (*n* = 3). (C) T_half_ values from FRAP experiments using BODIPY-C12 to label membranes showing that 200 μM PA leads to decreased membrane fluidity in HEK293 cells where AdipoR2 has been inhibited by siRNA, but that cocultivation with NT siRNA-treated cells in neighboring quadrants prevents this rigidification (*n* ≥ 10 cells). (D) FRAP curves for AdipoR2 siRNA-treated cell cultivated alone or cocultivated with NT siRNA-treated cells in neighboring quadrants (*n* ≥ 10 cells). (E) Quantitative PCR showing that SCD siRNA lowers SCD messenger RNA levels to <10% of that in the sample treated with NT siRNA (*n* = 3). (F) T_half_ values from FRAP experiments showing that 200 μM PA leads to decreased membrane fluidity in HEK293 cells where SCD has been inhibited by siRNA, but that cocultivation with NT siRNA-treated cells in neighboring quadrants prevents this rigidification (*n* ≥ 10 cells) Blue bars in 5C and 5F indicate cells treated with NT siRNA, yellow bars in 5C refers to AdipoR2 siRNA and red bars in 5F to SCD siRNA. ** *P* < 0.01 and *** *P* < 0.001.

We previously showed that the human AdipoR1 and AdipoR2 proteins are functional orthologs of the *C. elegans* PAQR-2: they regulate membrane composition and fluidity in HEK293 cells (derived from human embryonic kidneys) and are necessary to prevent membrane rigidification by the SFA PA (Devkota *et al.* 2017). We now examined whether the cell nonautonomous regulation of membrane homeostasis may also be conserved between *C. elegans* and humans. For this purpose, we made use of four-quadrant partitioned culture dishes to cultivate HEK293 cells in serum-free conditions but containing albumin, and used siRNA to inhibit the human AdipoR2 in some quadrants, then removed the partition and later measured membrane fluidity of cells in all quadrants using FRAP. Note that the FRAP method is reliable in mammalian cells: it has been used in numerous studies (Reits and Neefjes 2001; Staras *et al.* 2013), it documents membrane rigidity that correlates with SFA content in HEK293 cells (Devkota *et al.* 2017), and it matches results obtained with a separate method, namely the Laurdan dye method (Owen *et al.* 2011) (Figure S3, A–F). The results show that AdipoR2-expressing cells can act at a distance to maintain membrane fluidity in remote PA-challenged cells where AdipoR2 is silenced (Figure 5, A–D). Inhibiting the SCD gene in HEK293 cells also impairs their ability to prevent rigidification by PA, which is consistent with SCD being a downstream target of AdipoR2 (Figure 5, E and F), just as Δ9 desaturases act downstream of PAQR-2 in *C. elegans*. Additionally, SCD-expressing cells are also able to cell nonautonomously prevent membrane rigidification by PA in distant cells where SCD has been silenced (Figure 5, E and F). It therefore seems likely that AdipoR2-positive cells respond to the rigidifying PA challenge in a SCD-dependent manner to produce fluidizing UFAs that they can share with distant cells with which they have no physical contact. Note that the serum-free culture conditions used are likely stressful to these cells adapted to cultivation in the presence of serum (which contains cholesterol), and that the use of PA likely causes other effects besides membrane rigidification, including ER stress and inflammation (Peter *et al.* 2009). We therefore sought to more directly test whether mammalian cells can exchange lipids over significant distances and without cell-cell contact. Using transwell plates, we found that donor cells pretreated with ^3^H-labeled PA are able to deliver it to naïve acceptor cells from which they are separated by a membrane barrier, and that this transport is dependent on the presence of albumin or whole serum in the culture media (Figure S3, G–L).

## Discussion

**Figure 6:**
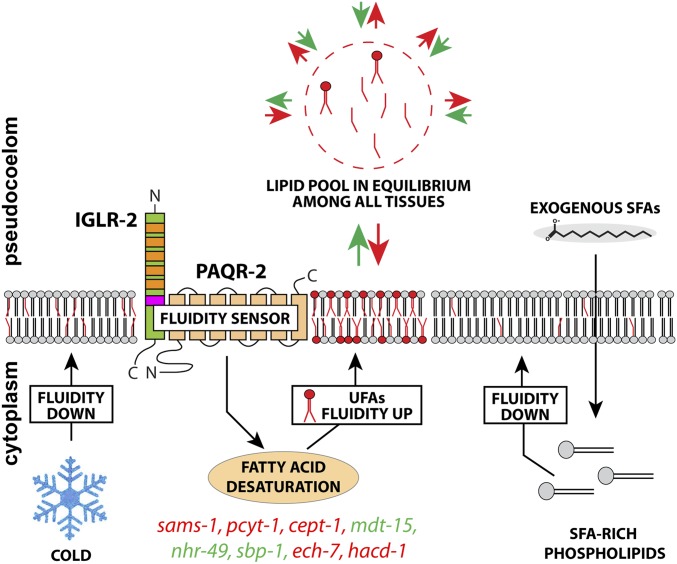
Updated model of membrane fluidity by PAQR-2/IGLR-2. From previous work, we know that the PAQR-2/IGLR-2 complex acts as a fluidity sensor that can promote fatty acid desaturation in response to membrane rigidification by low temperature or a diet rich in saturated fatty acids (Svensk *et al.* 2013, 2016; Devkota *et al.* 2017). The gene in colored types can suppress *paqr-2* and *iglr-2* mutant phenotypes as loss-of-function mutants (red) or gain-of-function mutants (green) that promote fatty acid desaturation (Svensk *et al.* 2013). This work suggests that large membranous tissues expressing the PAQR-2/IGLR-2 complex can contribute to systemic membrane homeostasis and that the mechanism likely relies on the exchange of lipids among most/all tissues in *C. elegans*. Signaling from *paqr-2*–positive cells may also contribute to cell nonautonomous membrane homeostasis, as mentioned in the *Discussion*.

Our extended mosaic analysis and tissue-specific expression experiments demonstrate convincingly that *paqr-2* and *iglr-2* can function cell nonautonomously and systemically when expressed in large tissues such as the hypodermis, gonad sheath, intestine, and body wall muscles. Our observations suggest a model where the PAQR-2/IGLR-2 complex can sense membrane rigidity in any large membranous tissue to induce local production of UFAs that are then shared with the rest of the worm (Figure 6). This model presumes extensive flux of fatty acids among all tissues and thus that, for example, expression of the PAQR-2/IGLR-2 complex specifically in the hypodermis will cease promoting fatty acid desaturation only when fluidity is normalized throughout the entire worm. We have one strong experimental support for this model: hypodermis-specific *paqr-2* expression, but not intestine-specific *paqr-2* expression, requires *fat-6* for systemic rescue of glucose tolerance (Figure 3, G and H). Because *fat-6* is the only desaturase expressed in the hypodermis (Brock *et al.* 2006), this result suggests that UFA production in the hypodermis is responsible for the systemic rescue in worms expressing *paqr-2* only in the hypodermis, a site of fat storage (Hellerer *et al.* 2007; Zhang *et al.* 2010) . This conclusion is further strengthened by the fact that hypodermis-specific expression of both *paqr-2* and *fat-6* is sufficient to rescue all phenotypes of the *paqr-2;fat-6* double mutant (Figure S2, C–E). The transport of lipids among *C. elegans* tissues is likely mediated via the pseudocoelomic fluid and may involve vitellogenins, other lipoprotein-like proteins such as DSC-4, or other pathways (Branicky *et al.* 2010). That lipids are in constant flux among *C. elegans* tissues should come as no surprise given that nearly 80% of phospholipids are replaced daily in an adult *C. elegans*, mostly using dietary fatty acids as building blocks, and thus implying extensive trafficking between the intestine and other tissues (Dancy *et al.* 2015).

Although the evidence is rather strong that phospholipids can be exchanged among *C. elegans* tissues, it is possible that, at least in some cases, signaling also contributes to systemic membrane homeostasis. For example, the PAQR-2/IGLR-2 complex could sense membrane rigidity in one tissue then signal to the intestine to promote fatty acid desaturation in that organ. The intestine would, in turn, provide more UFAs to all cells of the worm, presumably via the pseudocoelom. Speculating further, it is possible that activation of the presumed PAQR-2 hydrolase activity (Holland *et al.* 2011; Pei *et al.* 2011; Vasiliauskaité-Brooks *et al.* 2017) in large membranes causes the production of a signaling molecule that can diffuse to the intestine where it promotes fatty acid desaturation. One merit of this hypothesis is that it relies on the intestine for the production and distribution of fatty acids, a well-documented role for this organ. Signaling between the intestine and other tissues has also been documented. For example, the gonad is able to communicate to the intestine to regulate vitellogenin production (Balklava *et al.* 2016) whereas the intestine can regulate germline sex determination via specific signaling fatty acids (Tang and Han 2017). Similarly, the hypodermis can modulate lipid accumulation in the intestine via a *sma-3* dependent signal (Clark *et al.* 2018). This signaling hypothesis may explain why expression of *paqr-2* and *fat-6* did not rescue the fertility in the *paqr-2;fat-6* double mutant: systemic *paqr-2*-dependent membrane homeostasis from the gonad sheath cells may depend on a signal reaching *fat-6*–expressing tissues such as the hypodermis or intestine.

Our results with human HEK293 cells show that the cell nonautonomous nature of membrane homeostasis regulation is evolutionarily conserved from nematodes to mammals. Specifically, we showed that the membrane fluidity regulator AdipoR2 can promote membrane fluidity in distant AdipoR2-deficient cells and that this depends on the expression of the Δ9 desaturase SCD in the AdipoR2-expressing cells. This, as in *C. elegans*, suggests that cells are able to share membrane components, including fatty acids, over long distances and without cell-to-cell contact. While the most direct way for phospholipids exchange between cells or cellular compartments is through membrane contact sites (Jain and Holthuis 2017), several contact-independent mechanisms also exist. That lipoproteins and albumin can mediate the transport of fatty acids and phospholipids in processes involving phospholipid exchange proteins is an old observation that was the subject of reviews dating as far back as 1974 (Wirtz 1974). Albumin, which was present in our HEK293 serum-free cultures and likely accounts for the sharing of lipids between distant cells (see Figure S3, G–L), is the major long-range transport system for unesterified fatty acids, which make up <10% of blood fatty acids (van der Vusse 2009). Fatty acid esters, which make up >90% of blood fatty acids (*i.e.*, triacylglycerol, cholesterol esters, and phospholipids), are transported in blood by lipoprotein particles homologous to the *C. elegans* vitellogenins. These particles are made of a core consisting of a droplet of triacylglycerols and/or cholesteryl esters and a surface monolayer of phospholipid, cholesterol, and specific proteins (apolipoproteins), *e.g.*, apolipoprotein B-100 in the case of LDL.

An additional interesting finding in this study was the detection of several germline defects (vitellogenin uptake defect, increased apoptosis, defects in membranes of maturing oocytes), which all likely contribute to the low brood size of the *paqr-2* and *iglr-2* mutants. The accumulation of VIT-2::GFP in the pseudocoelom of the *paqr-2* and *iglr-2* mutants likely reflects a defect of the gonad to take up vitellogenins; this defect leads to increased production of vitellogenins by the intestine because of feedback mechanism between the germline and intestine (Balklava *et al.* 2016). This does not necessarily imply a primary gonad sheath membrane defect: others have shown that omega-6 PUFAs are essential for germline development (Chen *et al.* 2016) and to regulate the balance of lipid stores between the soma and germ cells (Lynn *et al.* 2015), and their production, or that of other regulatory lipids, may be defective in the *paqr-2* and *iglr-2* mutants.

The most important finding of this study is that membrane homeostasis is achieved cell nonautonomously both in a whole organism (*C. elegans*) and among cultivated human cells. Physiologically, there are clear benefits to cell nonautonomous maintenance of membrane homeostasis, including achieving “ordered heterogeneity,” a fundamental property of biological systems whereby tissues achieve healthy homogeneity even though they are composed of heterogeneous cells experiencing various levels of stresses or mutations (Rubin 2006). It will be interesting to explore the relevance of these findings for human physiology. For example, tumor cells with abnormal lipid metabolism could affect the membrane composition of neighboring healthy cells. Conversely, the cell nonautonomous nature of membrane homeostasis may contribute to the robustness of whole organisms in response to dietary or disease-state challenges, with impaired tissues or cells continuing to have functional membranes because of healthy neighbors.
